# Increased protein intake and corresponding renal acid load under a concurrent alkalizing diet regime

**DOI:** 10.14814/phy2.12851

**Published:** 2016-07-12

**Authors:** Thomas Remer, Jonas Esche, Danika Krupp

**Affiliations:** ^1^ DONALD Study DortmundDepartment of Nutritional EpidemiologyInstitute of Nutrition and Food Science (IEL)University of BonnDortmundGermany

We read with great interest the recently published article in *Physiological Reports* on protein intake, renal acid load, and hemodynamic responses (Teunissen‐Beekman et al. 2016). In their study, Teunissen‐Beekman et al. examined effects of an increase in protein intake (60 g/day, given as a mix of protein isolates, 20% pea, 20% soy, 30% egg‐white, 30% milk) on glomerular filtration rate (GFR) and related renally relevant parameters in overweight individuals with untreated elevated blood pressure and normal kidney function. Contrary to the author's initial hypothesis, neither fasting nor postprandial GFR values (the latter adjusted for the fasting measurements) were higher in the protein‐supplemented group (PROT) compared with a second group (MALT) which instead received 60 g/day of maltodextrin on an isoenergetic basis. The authors reported overall remarkably high GFR values in their subjects along with a short temporary GFR decline within 30 min postprandially in the protein‐supplemented group that was not seen in MALT.

As expected, the authors found a significantly higher renal acid load after 4 weeks on the protein supplement compared to 4 weeks on maltodextrin. 24‐h renal acid load was determined as urinary PRAL (uPRAL), a biomarker that characterizes the mineral component (including sulfate from sulfur‐containing amino acids) of total renal net acid excretion (NAE) without considering the organic acid component (Krupp et al. 2014). Unexpectedly, the observed uPRAL difference after 4 weeks on higher protein ingestion versus baseline was only about 15 mEq/day. This is half of what is known from the literature for protein increases of that magnitude (Remer and Manz 1994, 1995a). In accord herewith, in a separate article on the present diet experiment (Teunissen‐Beekman et al. 2012), the authors reported differences in urinary sulfate excretion after 4 weeks (for PROT vs. MALT, and for PROT vs. baseline) of 7–8 mmol/day corresponding to 15 mEq of renally excreted dietary acidity stemming from sulfur amino acid metabolism. However, 30 mEq/day of “sulfate acidity” would have been expected with an increase of 60 g/day of a protein mix of that composition (Remer and Manz 1995b). It appears worth considering, whether a lower compliance at the end of the 4 week observation period might have contributed to the too low acid excretion after protein supplementation of that magnitude.

Interestingly, also the average renal acid load component (4) measured as uPRAL in 24‐h urine samples of both groups (PROT and MALT) has been found to be remarkably low, both at baseline and after 4 weeks of diet intervention. Mean uPRAL varied between ca. −5 mEq and ca. −20 mEq/day (Figure 4; Teunissen‐Beekman et al. 2016) confirming a largely alkalizing basic nutrition present already at run‐in. In contrast, healthy adults of the DONALD study with an average protein intake of 115 g/day – that is, almost comparable to PROT – show mean uPRAL values around +50 mEq/day corresponding to a NAE of ca. 90 mEq/day and reflecting a rather higher dietary acid load. The fact that the authors, notwithstanding, found a low uPRAL range throughout (<0 mEq/day), also in PROT after a mean protein intake of ca. 130 g/day (i.e., with the 60 g protein supplement added) strongly suggests that nutritional counseling of the study participants had successfully led to a nutrition mostly rich in base equivalents. This is also supported by the decrease of uPRAL from ≈ −7 mEq/day to −23 mEq/day in the maltodextrin group after 4 weeks versus baseline (Figure 4). Such a rather alkaline diet (possibly also including sodium bicarbonate‐containing beverages) would also explain why Teunissen‐Beekman et al. (2016) did not observe significant changes in blood pH and bicarbonate with the high‐protein diet.

All in all, the findings of Teunissen‐Beekman et al. (2016) underline the importance of increasing dietary alkali equivalents, that is, low‐PRAL foods, particularly if protein intake is raised, so that the protein‐related dietary acidity can be effectively neutralized. Also, the conclusions of Teunissen‐Beekman et al. that no postprandial changes in blood pH or bicarbonate are to be expected with increase of around 60 g/day in daily protein intake, cannot be generalized to more typical subjects eating more typical Western diets with a higher PRAL or having a more typical age‐specific GFR.

## Conflict of Interest

The authors have nothing to disclose.
